# Supplementary material for: Screening and Analysis of Possible Drugs Binding to PDGFRα: A Molecular Modeling Study
Source: Int J Mol Sci. 2023 Jun 1;24(11):9623. doi: 10.3390/ijms24119623 (PMC10253372; doi:10.3390/ijms24119623)
Supplement: Supplementary file 1 [file ijms-24-09623-s001.zip › ijms-2283730-supplementary Figure S1 and Table legend.pdf]

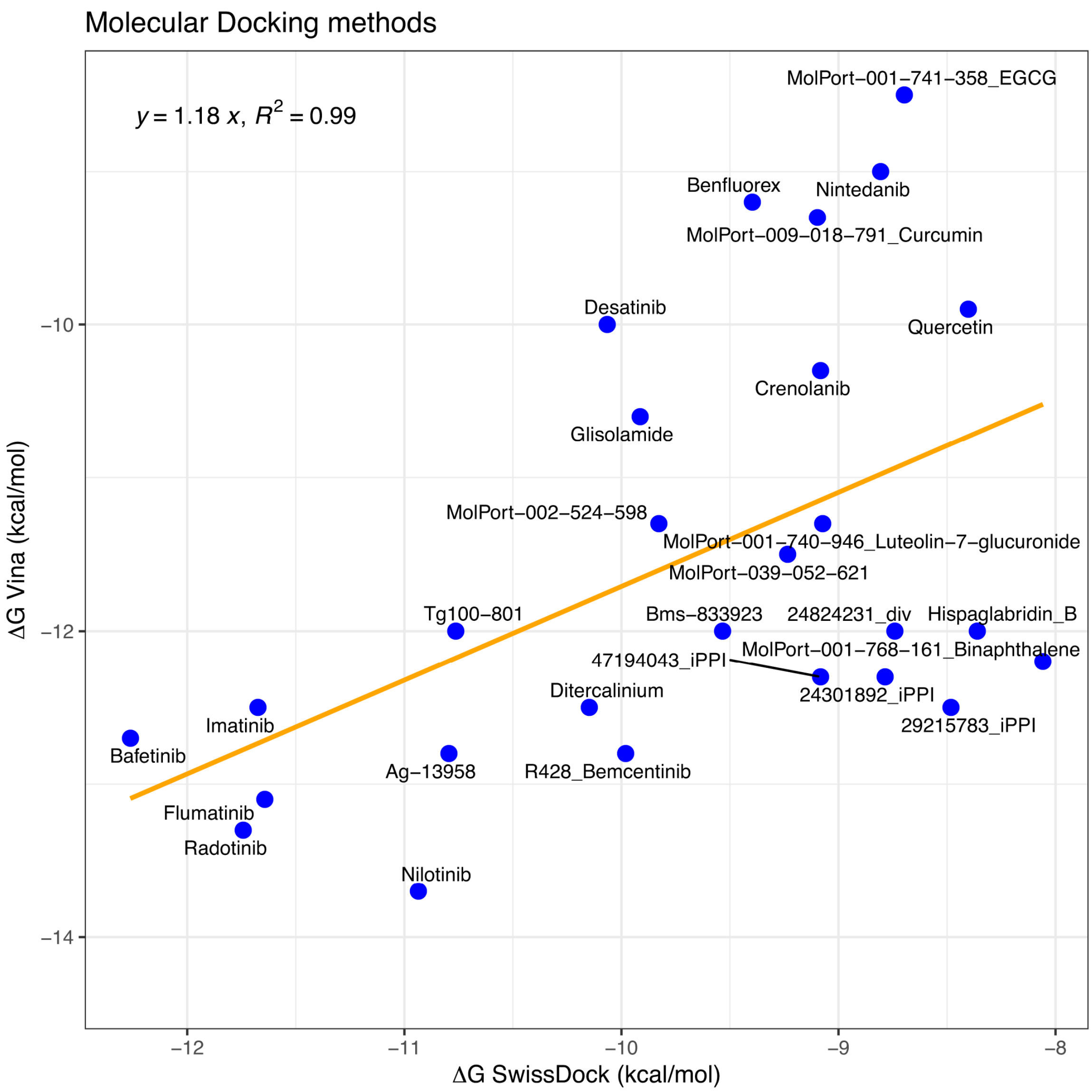


**Figure S1:** Correlation between the two molecular docking algorithms used (Autodock Vina-based docking genetic algorithm and EADock DSS algorithm).

**Tables S1–S5.** List of compounds included in the libraries used for structure-based virtual screening against the human intracellular PDGFRα.
